# Construction of the panoptosis-related gene model and characterization of tumor microenvironment infiltration in hepatocellular carcinoma

**DOI:** 10.32604/or.2023.028964

**Published:** 2023-06-27

**Authors:** XINRUI SHI, XIA GAO, WENCONG LIU, XUEJIAO TANG, JIAYI LIU, DONGCHEN PAN, XUEQING DUAN, YUQING JIN, WEIYAN REN, LEI YANG, WENXUAN LIU

**Affiliations:** 1 Hebei Key Laboratory of Environment and Human Health, School of Public Health, Hebei Medical University, Shijiazhuang, China; 2 Department of Ultrasonics, The First Affiliated Hospital of Hebei Medical University, Shijiazhuang, China

**Keywords:** PANoptosis, Hepatocellular carcinoma, Prognosis, Single-cell analysis, Immune infiltration

## Abstract

Hepatocellular carcinoma (HCC) is the most common fatal cancer worldwide, patients with HCC have a high mortality rate and poor prognosis. PANoptosis is a novel discovery of programmed cell death associated with cancer development. However, the role of PANoptosis in HCC remains obscure. In this study, we enrolled 274 PANoptosis-related genes (PANRGs) and screened 8 genes to set up a prognostic model. A previous scoring system calculated PANscore was utilized to quantify the individual risk level of each HCC patient, and the reliability of the prognostic model has been validated in an external cohort. Nomogram constructed with PANscore and clinical characteristics were used to optimize individualized treatment for each patient. Single-cell analysis revealed a PANoptosis model associated with tumor immune cell infiltration, particularly natural killer (NK) cells. Further exploration of hub genes and assessment of the prognostic role of these 4 hub genes in HCC by quantitative real-time PCR (qRT-PCR) and immunohistochemistry (IHC). In conclusion, we evaluated a PANoptosis-based prognostic model as a potential prognostic biomarker for HCC patients.

## Introduction

Hepatocellular carcinoma (HCC) is currently one of the most common cancers worldwide and is regarded as the third most frequent cause of cancer-related deaths worldwide [1,2]. HCC accounts for approximately 75%–85% of all primary liver cancer cases and is characterized by high heterogeneity, poor prognosis, and drug response [3–5]. Currently, only a few treatments are available for HCC, and the survival advantage of chemotherapy is not apparent for advanced patients [6]. Although there are many biomarkers or genetic models that have been found to potentially predict the prognosis of HCC patients, few of them can be applied in clinical practice. Therefore, the development of a validated model to predict the prognosis of HCC patients is essential to provide new clinical management guidance.

PANoptosis is a newly described form of inflammatory cell death that encompasses three major pathways of programmed cell death (PCD), including pyroptosis, apoptosis, and Necroptosis, and there is extensive crosstalk between these molecular processes. PANoptosis was previously shown to be controlled by the cytoplasmic polyprotein complex PANoptosome. When one or more PCDs are blocked, it is replaced by alternative death mechanisms. Apoptosis is executed by the promoter caspases caspase-8/10 or -9 downstream of caspase-3 and -7. The resistance of cancer cells to apoptosis may be an essential mechanism leading to cancer [7]. Pyroptosis is performed by members of the gasdermin family and is characterized by cell swelling, plasma membrane rupture, and cytokine release [8]. Necroptosis is a RIPK1- and RIPK3-dependent anti-tumor immune mechanism [9]. Previous studies have shown that PANoptosis plays a critical role in many diseases, such as microbial infection, inflammation, and malignant tumor [10–12]. Moreover, PANoptosis is presently considered to be associated with tumor growth and metastasis, drug resistance, and poor prognosis.

Growing evidence has indicated that gene signatures based on pyroptosis, apoptosis, and necroptosis are associated with the progression and prognosis of HCC patients [13–15]. The relationship between PANoptosis and immune regulation, however, has remained unclear.

This study aims to establish a prognostic model in the TCGA database. Previous studies have explored the relationship between PANoptosis and cancer and developed a quantitative indicator that represents PANoptosis-related risk score named PANscore [16] to predict the outcome of HCC patients with prognosis. This study constructed PANscore in HCC based on this foundation to further explore the potential biological patterns of PANoptosis. Furthermore, Nomogram was developed to effectively predict prognosis and help select patients for individualized treatment. Meanwhile, our risk prediction model verified that four key genes (DAP3, PPP2R5B, GSDME, and PLK1) were overexpressed in HCC cell lines. Finally, immunohistochemical results proved that DAP3, PPP2R5B, GSDME, and PLK1 are potential prognostic biomarkers for HCC.

## Materials and Methods

### Sources and acquisition of data and samples

#### Datasets sources and preprocessing

We obtained RNA-sequencing (RNA-seq) data and the relevant clinical infor-mation of 374 liver cancer samples and 50 normal liver samples from the Cancer Ge-nome Atlas (TCGA, https://portal.gdc.cancer.gov/repository) database. Gene expression profiles were normalized by using the scale method provided in the “limma” R package. In this study, the overall survival (OS) values of the HCC patients who were missing were excluded. In addition, transcriptomic and clinical data were downloaded from the International Cancer Genome Consortium (ICGC, https://dcc.icgc.org/projects/LIRI-JP) on the clinical characteristics of 243 HCC patients and 202 normal individuals (LIRI-JP). Information on HCC patients and normal samples were obtained from Japan, and the gene expression values were normalized. 336 Metabolism-related gene sets were obtained from Gene set enrichment analysis (GSEA, https://www.gsea-msigdb.org/gsea/).

The hepatocellular carcinoma scRNA raw sequence reads CNP0000650 were downloaded from the China National GeneBank DataBase (CNGBdb, https://db.cngb.org/). Includes 16,498 full-length single-cell transcriptomes from 18 patients with primary or early relapsed HCC.

#### Clinical sample acquisition and ethics statement

We retrospectively collected tissues from 44 HCC patients who underwent surgical resection at the Fourth Hospital of Hebei Medical University (from December 2009 to March 2015). These patients were followed up until 2022. All patients had resectable HCC and did not receive preoperative anticancer treatment. The ethical review of this study protocol was approved by the Ethics Committee of Hebei Medical University. All patients were informed and signed an informed consent form before participating in the study. The experimental protocol was approved by the Ethics Committee of Hebei Medical University (approval number: [P] 2022077).

#### Cell lines

The HepG2 human HCC cell line used in this study was obtained from Wuhan Sevier Biotechnology Company, and human HCC HL-7721 cells and human normal hepatocyte line L-O2 were retained by our laboratory.

### Acquisition of the PANoptosis gene list and identification of prognostic DEGs

We created a list containing 274 PANoptosis related genes (PANRGs) from previous research and the literature [17–19], and the details of all the genes are shown in Suppl. Table S1. The list was combined with the apoptosis, pyroptosis, and necroptosis gene sets, and the overlapping part of the genes was removed.

The RNA-seq data in TCGA were normalized to fragments per kilobase million (FPKM). The “limma” package (version 3.52.4) was used to identify the DEGs between the tumor and paired adjacent nontumor tissues. The threshold value was set to |logFC| > 1 and FDR < 0.05. Univariate Cox regression analysis identified PANRGs with a potential prognosis, and genes with a *p* < 0.05 were selected. The gene interaction network was constructed using the STRING (STRING, https://string-db.org/) database.

### Construction of the PANoptosis-related gene prognostic model

In the TCGA datasets, 374 HCC patients with survival information were taken as the training set. To avoid overfitting the prognostic signature, the “glmnet” package (version 4.1-4) was used to perform the least absolute shrinkage and selection operator (LASSO) regression to construct a prognostic model. Then, 8 prognostic PANoptosis-related differentially expressed genes (DEGs) were identified for further analysis. The estimated regression coefficient was weighted and combined with the PANRG expression value. The PANscore formula for the progno-sis-related gene signature revealed the following: PANscore = coefficient of gene (1) * expression of gene (1) + coefficient of gene (2) * expression of gene (2) +…+ coefficient of gene (n) * expression of gene (n).

The PANscore for each HCC patient was calculated based on the above formula. Patients can be divided into two groups (high PANscore group and low PANscore group) by median PANscore. In addition, we performed Principal Components Analysis (PCA) and t-Distributed Stochastic Neighbor Embedding (t-SNE) to explore the distribution of the different groups. Furthermore, to explore the correlation between the PANoptosis-related score and the overall survival of HCC patients, the “survival” package (version 3.4-0) was used to perform survival analysis. To further validate the PANoptosis-related scores, we constructed time-dependent receiver operating characteristic (ROC) curves with the “survivalROC” package (version 1.0.3) to evaluate the predictive power of PANscore.

The validation dataset LIRI-JP cohort was used to assess the performance and prognostic power of the PANoptosis-related score using time-dependent ROC analysis and Kaplan-Meier log-rank tests.

### Functional enrichment analysis

The R packages “clusterProfiler” (version 4.4.4) and “ggplot2” (version 3.3.6) were used for GSEA (gene set en-richment analysis), including the Gene Ontology (GO) and Kyoto Encyclopedia of Genes and Genomes (KEGG) databases. The analysis for the high PANscore group and low PANscore group based on DEGs was set to |log2FC| ≥ 1, FDR < 0.05. *p* values were adjusted using the BH method. Single sample gene set enrichment analysis (ssGSEA) was performed with the GSVA R package (version 1.44.5) to calculate the infiltration score of 16 immune cells and the activity of 13 immune-related pathways. Subsequently, advanced Heatmap Plot was performed using the OmicStudio tools at https://www.omicstudio.cn.

### Analysis of infiltrating immune cells in HCC

The association between the prognostic model and immunocyte infiltration was evaluated by the CIBERSORT algorithm [20], which was used to calculate the proportion of each kind of tumor-infiltrating immune cell, such as B cells, CD4+ T cells, CD8+ T cells, neutrophils, macrophages, and dendritic cells. in HCC patients. The *p* value threshold was set to 0.05. Subsequently, we estimated the differences in the tumor immune microenvironment between the two groups by evaluating the expression of each type of immune cell in the high PANscore group and low PANscore group.

### Nomogram and calibration

With the R package “rms” (version 6.3-0), a nomogram was constructed to predict OS rates at 1, 3, and 5 years. The predictors (TNM stage and PANscore) were employed. Correction curves based on the Hosmer-Lemeshow test were applied to illustrate the uniformity between the practical outcome and the model prediction model.

### Single-cell RNA sequence clustering for dimension reduction

To further dissect the tumor immune microenvironment in HCC tissues at the single-cell level, we utilized previously published single-cell RNA (scRNA) data from 12 primary liver cancer and 6 early relapsed HCC patients with tumor specimens and paired adjacent nontumor tissues [21]. A total of 16,498 single-cell transcriptome data were included. First, cell filtration of scRNA data was processed by the R package “Seurat” (v4.2.0) [22]. Then, the “ScaleData” function was used to scale all genes, and the “RunPCA” function was used to conduct PCA for highly variant genes to reduce the dimensionality. In addition, we clustered the cells by the “FindNeighbors” and “FindClusters” functions (dim = 20, solution = 1) to find cell clusters and further reduce the dimensionality by the UMAP method. After finding marker genes the automatic cell annotation R package, signleR (version 2.0.0) is used to annotate the cell type.

### Screening for PANoptosis-related key genes

To identify key prognostic genes correlated with PANoptosis in HCC, weighted gene coexpression network analysis (WGCNA) was used to construct key modules based on RNA-seq data of 374 tumor samples and 50 normal samples from the TCGA cohort. First, a suitable power exponent was employed to construct the weighted gene network. Hierarchical clustering analysis was performed. Then, the branches of the clustering tree and colors indicated the different gene modules. Furthermore, we calculated the correlation between gene modules and PANscores and identified a module associated with PANscores. The module with the strongest positive correlation with PANscores was selected for subsequent analysis. Key genes were identified by the intersection of module genes with PANRGs.

### Immunohistochemistry (IHC)

Expression of PLK1 in tumors and adjacent normal tissues was detected using IHC. PLK1 rabbit anti-human antibody (Cell Signaling Technologies, Danvers, Massachusetts, USA) was employed. The dilution concentration was 1:500, and all sections were independently interpreted by two pathologists who kept clinical information confidential. All samples were routinely fixed, paraffinembedded, serially sectioned, and immunohistochemically stained. Immunohistochemical staining was scored using the H-Score method [23]. Immunohistochemical staining kits (Beijing Bioson Biotechnology Company, Beijing, China) are used according to the instructions. After the staining was completed in the tissue microarray scanner for automatic scanning, the software automatically scored each tissue staining with a scale of H-Score.

$$\begin{array}{l} H\text{-}Score\;=\;\;\left(\%\;unstained\;tumor\;cells\;\times \;0\right)\\ \;+\;\left(\%\;weakly\;stained\;tumor\;cells\;\times \;1\right)\\ \;+\;\left(\%\;moderately\;stained\;tumor\;cells\;\times \;2\right)\\ \;+\;\left(\%\;strongly\;stained\;tumor\;cells\;\times \;3\right). \end{array}$$

### Total RNA extraction and qRT‒PCR

The human normal hepatocyte line LO2 and HCC cell lines HepG2 and HL-7721 were cultured in Dulbecco’s modified Eagle’s medium (DMEM, Gibco BRL, USA) containing 10% fetal bovine serum (FBS, zeta life, USA) in a 5% CO_2_ incubator at 37°C. Total cellular RNA was extracted using TRIpure (Aidlab, Beijing, CN). The procedure was performed according to the manufacturer’s instructions. Total RNA was extracted from liver tissue and hepatocyte lines with TRIZOL (TIANGEN, Beijing, CN) reagent. NanoDrop-2000 (Thermo Fisher Scientific, Waltham, MA, USA) was used to assess the RNA quality and concentration. cDNA was then obtained by reverse transcription with a cDNA Synthesis Kit (Thermo Fisher Scientific, Waltham, MA, USA), and qRT-PCR (TIANGEN, FP205, GER) was used for analysis. Finally, the mRNA expression was normalized with the GAPDH gene. The specificity of the qRT-PCR products was evaluated by observing whether the melting curve was a single peak. Three replicate wells were set up for each reaction, and those with Cq values not exceeding 0.5 between replicate wells were used for data analysis, in which the mean Cq values were calculated.

### Fluorescence microscopy to detect apoptosis, necroptosis, and pyroptosis

A YO-PRO-1 (YP1)- and propidium iodide (PI) cell death detection kit (C1075S, Beyotime, Jiangsu, China) was employed. LO2, HepG2 and 7721 cells were cultured on 6-well plates, and cells were stained after dilution with YP1 and PI reagents according to the instructions and incubated at 37°C for 20 min protected from light. The labeled cells were observed under a fluorescence microscope: YP1-positive cells (green) underwent apoptosis or necrosis, and PI-positive cells (red) underwent necroptosis, pyroptosis or ferroptosis.

### Pan-cancer analysis of the effect of key PANRGs on cell growth

To perform the analysis, the DepMap homepage (https://depmap.org/portal) was logged into, the key gene name was inputted, “CRISPR (DepMap 22Q2 Public)” was selected for the dataset, the results were downloaded, and the image was processed with Adobe Illustrator CC 2019.

#### Effect of key gene knockdown on the growth of hepatocellular carcinoma cells

Behan and his team published CRISPR‒Cas9 library screening data [24] (https://score.depmap.sanger.ac.uk/downloads). The Uniform Resource Locator (URL) was opened, and the “Download” option was clicked to download the data “broad_essentiality_matrices_190724.zip190724.zip”. Liver cancer cell lines were se-lected, and the effects of four key PANRGs on the growth of various cell lines were an-alyzed and plotted using GraphPad Prism 8.0. Hepatocellular carcinoma cell lines were selected, and the effect of key PANRG knockdown on the growth of hepatocellular carcinoma cells was analyzed using GraphPad Prism 8.0.2 Plot.

### Statistical analysis

All statistical analyses were performed in R 4.2.1 and GraphPad Prism 8.0.2. For samples that followed a normal distribution, Student’s *t*-test was used to compare differences in gene expression between different hepatocyte lines or different liver tissues. Groups of variables that did not follow a normal distribution were analyzed using a nonparametric test. *p* < 0.05 indicates statistical significance (two-sided).

## Result

### Identification of differentially expressed prognostic PANoptosis genes

**Figure 1:**
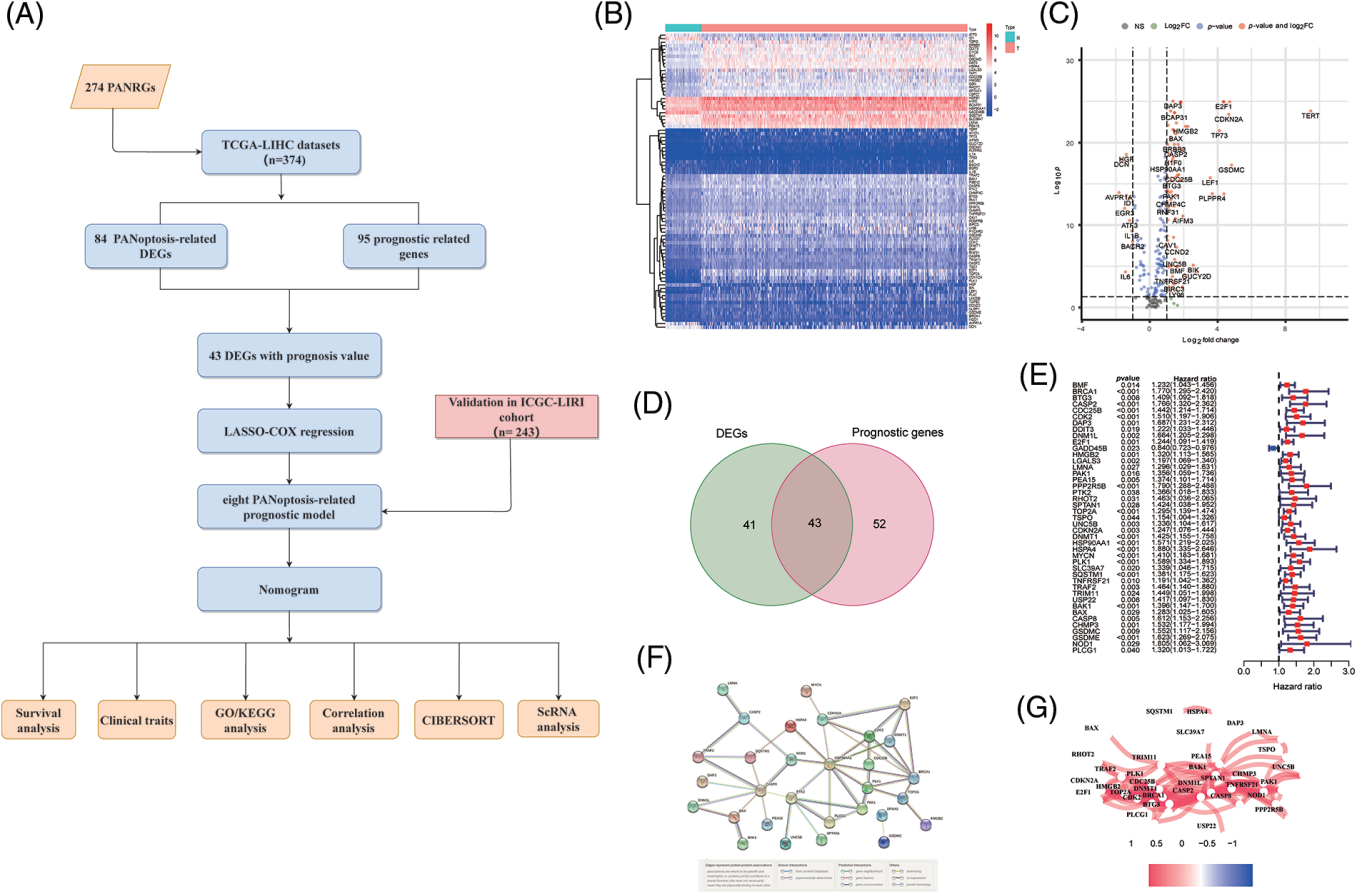
Screening PANRGs of HCC patients from TCGA database. (A) Flow chart of this study. (B) Heatmap of differential expression of PANRGs between normal and tumor groups. (C) Vol-cano plot of differentially expressed PANRGs. (D) Venn diagram identifies prognostic differences between tumor and adjacent normal tissue. (E) Univariate COX regression analysis of forest plots between PANRGs and OS. (F) A correlation network of 43 prognostic-related differentially ex-pressed genes. (G) Protein-protein interaction networks (PPI) interactions between progno-sis-related differentially expressed PANRGs.

The overall workflow of our work is presented in Fig. 1A. In this study, 374 HCC patients from the TCGA-LIHC cohort and 243 HCC patients from the ICGC-LIRI-JP cohort were included. The clinical characteristics of these HCC patients are summarized in Table 1.

**Table 1:** Clinical baseline information of HCC patients in this study

Variable		TCGA-LIHC	ICGC-LIRI-JP
Number of cases		371	260
Overall survival days (Median, IQR)		602 (346, 1094.5)	750 (510, 1050)
Censored (%)	Alive	239 (64.4)	214 (82.3)
	Dead	132 (35.6)	46 (17.7)
Age (median, IQR)		61 (51, 69)	69 (62, 74)
Gender (%)	Female	120 (32.3)	68 (26.2)
	Male	251 (67.7)	192 (73.8)
Grade (%)	G1	55 (14.8)	NA
	G2	178 (48.0)	NA
	G3	120 (32.3)	NA
	G4	13 (3.5)	NA
	Unknown	5 (1.3)	NA
Clinical stage (%)	Stage I	174 (46.9)	40 (15.4)
	Stage II	85 (22.9)	117 (45.0)
	Stage III	84 (22.6)	80 (30.8)
	Stage IV	4 (1.1)	23 (8.8)
	Unknown	24 (6.5)	0
T stage (%)	T1	184 (49.6)	NA
	T2	92 (24.8)	NA
	T3	79 (21.3)	NA
	T4	13 (3.5)	NA
	TX	1 (0.3)	NA
	Unknown	2 (0.5)	NA
M stage (%)	M0	269 (72.5)	NA
	M1	3 (0.8)	NA
	MX	99 (26.7)	NA
N stage (%)	N0	253 (68.2)	NA
	N1	4 (1.1)	NA
	NX	113 (30.5)	NA
	Unknown		

We enrolled 274 PANoptosis genes, including 156 apoptosis, 65 necroptosis and 53 pyroptosis genes, and 84 differentially expressed PANoptosis genes between tumor samples and noncancer samples were obtained (Figs. 1B and 1C). Subsequently, Univariate Cox regression analysis (*p* < 0.05) was employed to identify the 95 PANoptosis genes associated with prognosis in HCC patients. After overlapping differentially expressed genes and prognosis-related genes, 43 PANRGs were ultimately obtained for follow-up analysis (Figs. 1D and 1E). Except for GADD45B, the remaining 42 genes were defined as deleterious effect genes with all-hazard ratio (HR) values of >1. The interaction network and correlation network between these PANRGs are shown in Figs. 1F and 1G.

### Establishment of the eight-gene model based on PANRGs

**Figure 2:**
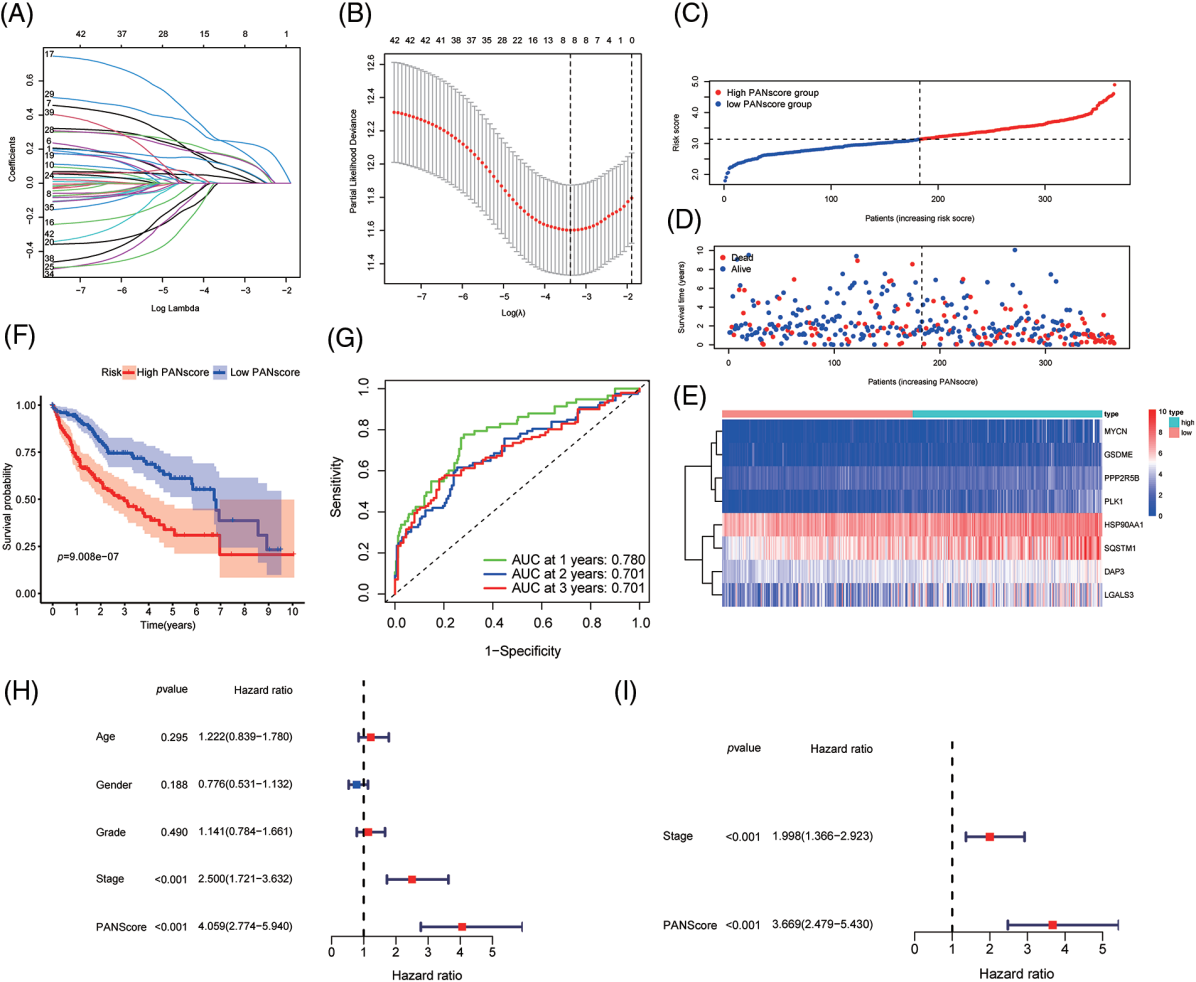
Development of PANoptosis-related prognostic models of HCC patients in TCGA co-hort. (A, B) The LASSO coefficient profiles and the penalty plots of the model for 43 prognostic genes. (C–E) Distribution of PANscore, survival status of HCC patients, and expression of eight risk genes related to PANoptosis. (F) Kaplan-Meier curves for OS of patients in the high PANscore and low PANscore groups. (G) The AUC of time-dependent ROC curves evaluates the predictive performance of PANscore. (H, I) Results of univariate and multivariate COX regression analysis of OS to evaluate the independence of PANscore.

To better predict the clinical outcomes of HCC, LASSO-penalized Cox analysis was conducted to further narrow down the mRNA expression profile based on the optimal value of λ (Figs. 2A and 2B). This algorithm compresses the coefficients of some less important variables to zero and keeps the coefficients of important variables greater than zero to reduce the number of covariates in the Cox regression. Finally, 8 genes were identified to construct PANoptosis risk prognostic models. The PANscore for each HCC patient was calculated as follows:

$$\begin{array}{l} PANscore=\;\;(0.0662\;\times \;expression\;value\;DAP3)\\ \;+(0.0305\;\times \;expression\;value\;LGALS3)\\ \;+(0.2286\;\times \;expression\;value\;PPP2R5B)\\ \;+(\;0.1022\;\times \;expression\;HSP90AA1)\\ \;+(0.1620\;\times \;expression\;value\;MYCN)\\ \;+(\;0.2467\;\times \;expression\;value\;PLK1)\\ \;+(0.1563\;\times \;expression\;value\;SQSTM1)\\ \;+(\;0.1022\;\times \;expression\;value\;GSDME) \end{array}$$

**Supplementary Figure 1:**
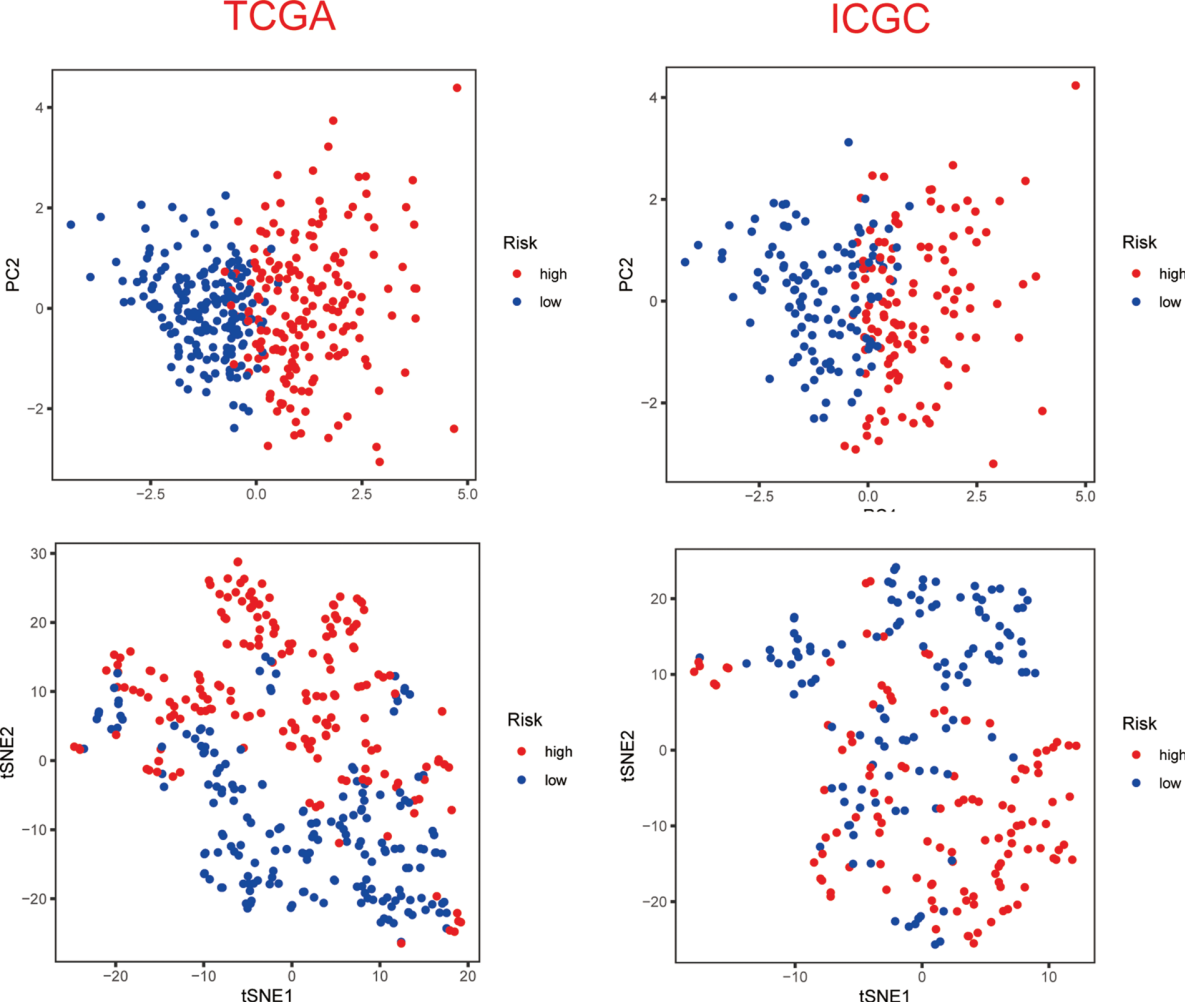
PCA and t-SNE analysis of ICGC dataset.

Based on the median value of the PANscore, HCC patients were divided into a high PANscore (n = 182) group and a low PANscore (n = 183) group (Figs. 2C–2E). Then, PCA and t-SNE analysis indicate that the samples in the high PANscore and low PANscore groups could be well distinguished (Suppl. Fig. 1). Moreover, the high PANscore group had a lower survival time and a higher probability of death. In addition, a heatmap of the 8 prognosis-related genes between the high- and low-PANscore groups is shown in Figs. 2C–2E. Survival analysis of the TCGA cohort demonstrated worse OS outcomes in the high PANscore group, and the predictive performance of the PANscore was assessed using a time-dependent ROC curve, the area under the curve (AUC) at 1-, 2-, and 3-year was up to 0.781, 0.701, and 0.701 (Figs. 2F and 2G). In addition, multivariate Cox regression analysis was performed for all variables that were significant in the univariate Cox regression analysis (*p* < 0.05) (age, sex, stage, and PANscore) of 350 HCC patients and to determine whether PANscores were independent prognostic predictors of OS. In the univariate and multivariate Cox analyses, stage and PANscore were the independent predictors of OS, and the PANscore significantly correlated with OS and had a high hazard ratio (HR) value (HR = 3.669, 95% CI = 2.497–5.430) (Figs. 2H and 2I).

### Validation of the PANscore in the ICGC cohort

**Figure 3:**
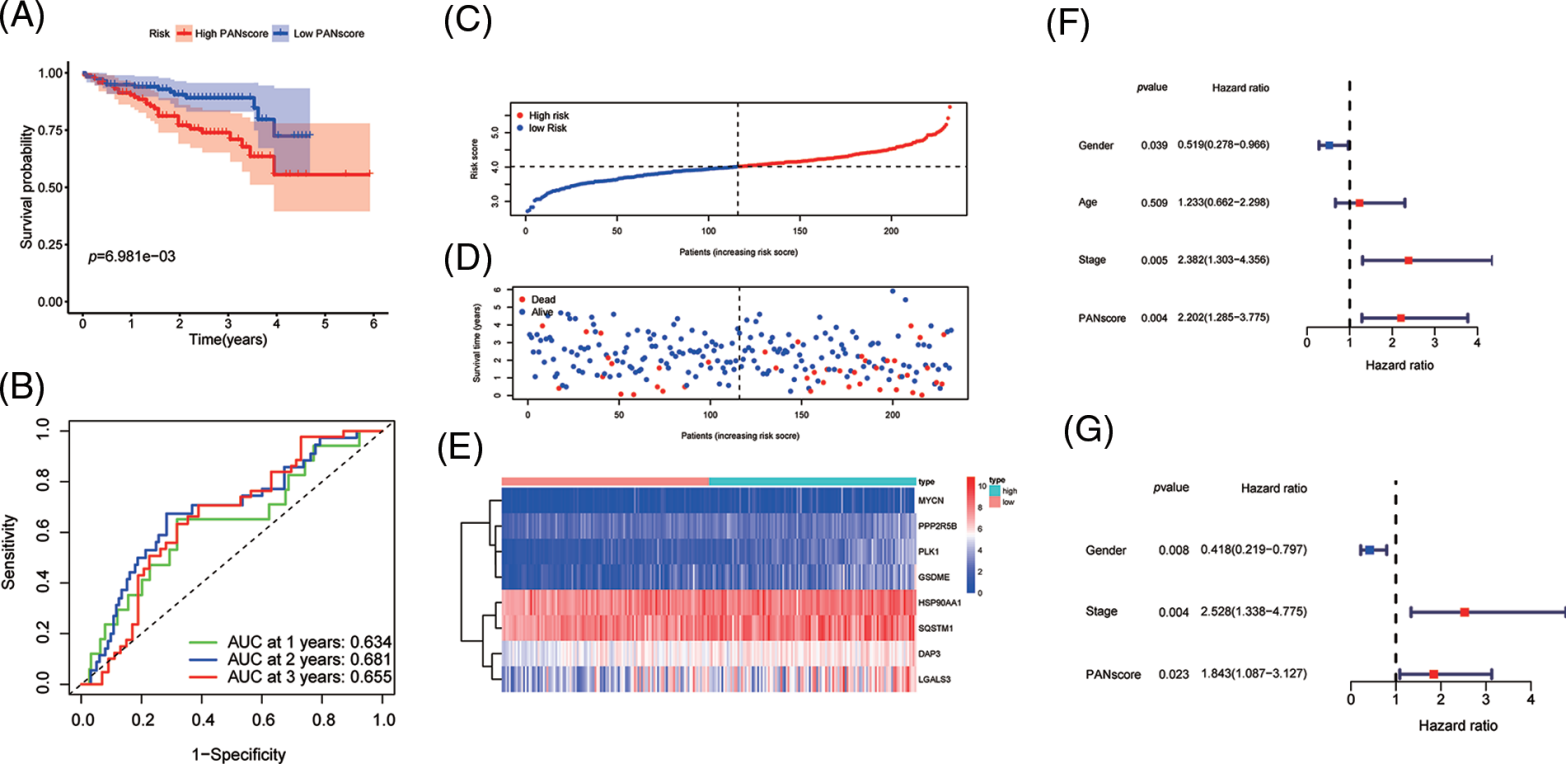
External validation of PANscore prediction performance based on ICGC cohort. (A) Kaplan-Meier curves in the high PANscore and low PANscore groups. (B) ROC curves to assess the predictive efficiency of PANscore in the ICGC cohort. (C–E) PANscore distribution, survival status, and heatmap of expression of 8 risk genes related to PANoptosis. (F, G) Forest plots of univariate and multivariate COX regression analysis results.

To validate the stability of the 8-gene model, the ICGC-LIRI-JP cohort was included as a validation cohort, to which a workflow similar to that of the training set was applied. The survival analysis revealed a statistically significant difference in the survival rates between the high- and low-PANscore groups (*p* < 0.001). In contrast, the overall survival in the high PANscore group was significantly worse than the low PANscore group (Fig. 3A). Moreover, the PANscore constructed in the TCGA cohort was used to calculate the area under the ROC curve (AUC), with values of 0.634, 0.681, and 0.655 for 1, 2, and 3 years, respectively (Fig. 3B). The distribution of risk scores and the associated survival status of individual patients with HCC are shown in Figs. 3C–3E. Similarly, in the ICGC cohort, 3 variables were independent prognostic predictors of OS, including sex, stage, and PANscore. PANscores are also significantly associated with HCC patient prognosis (*p* = 0.023, HR = 1.843, 95% CI = 1.087–3.127) (Figs. 3F and 3G). Therefore, the PANscore demonstrated favorable predictive ability in the TCGA and ICGC cohorts, and the PANscore based on PANRGs would be considered an independent predictor of OS for HCC patients.

### Functional enrichment analysis and tumor immune cell infiltration

To further explore the biological function of PANoptosis, we performed GSEA to find the GO and KEGG pathways enriched in differentially expressed genes with a threshold of 0.05.

**Figure 4:**
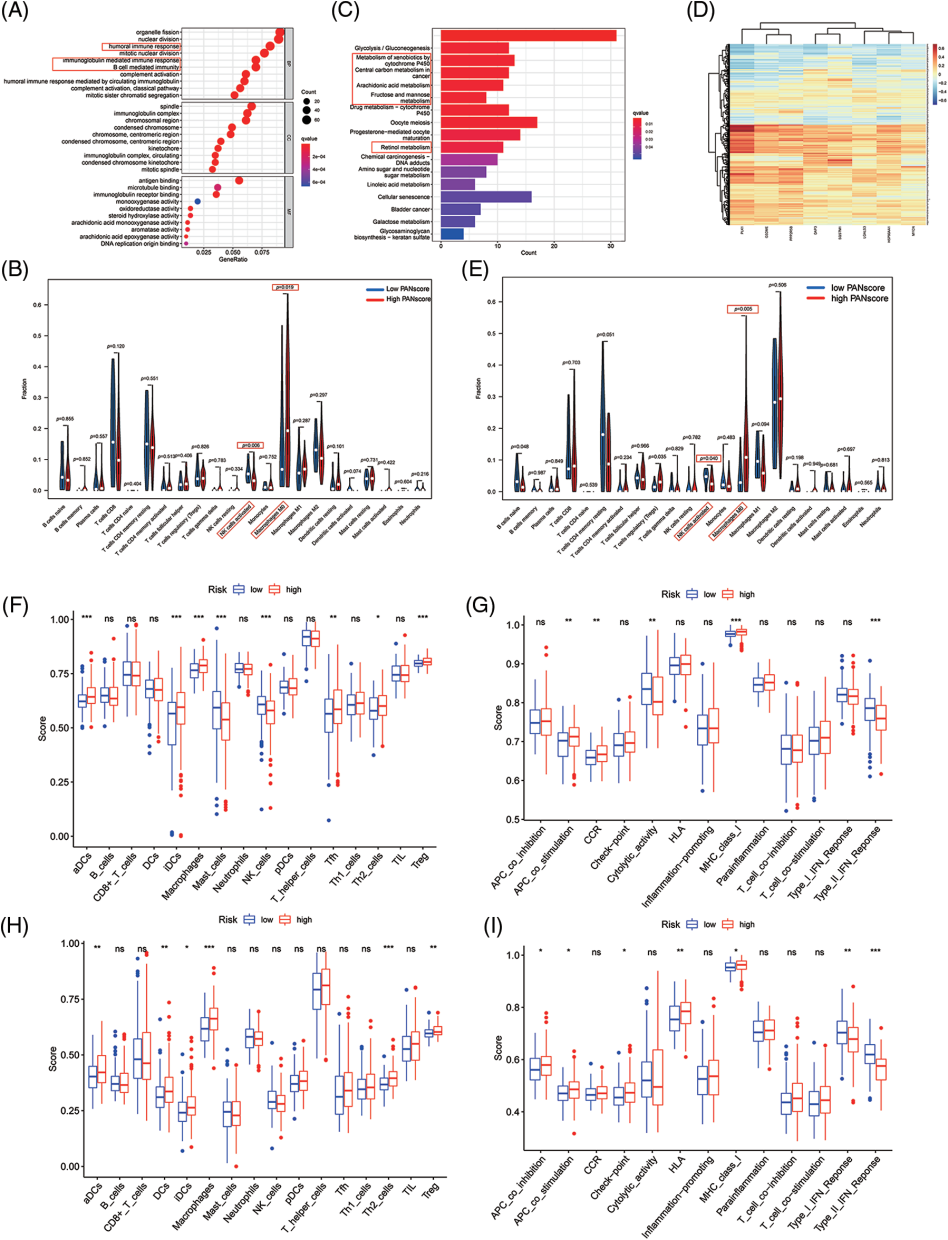
Enrichment analysis of PANscore-related DEGs. (A, C) GO enrichment and KEGG pathway in the TCGA cohort. (B, E) Violin plots of immune cell infiltration in the high PANscore and low PANscore groups in the ICGC dataset. (C) The violin plot of immune cell infiltration between the high PANscore and low PANscore groups. (D) Heatmap indicating the correlation between eight PRGs and metabolism-related genes. (E) Violin plots of immune cell infiltration in the high PANscore and low PANscore groups in the ICGC dataset. (F, H) Comparison of ssGSEA scores for different PANscore groups in TCGA cohort and ICGC cohort, box plot showing the scores of 16 immune cells. (G, I) ssGSEA scores of 13 immune-related functions in different PANscore groups in TCGA cohort and ICGC cohort. Adjusted *p* values are expressed as: ns, not significant *p < 0.05; **p < 0.01; ***p < 0.001.

**Supplementary Figure 2:**
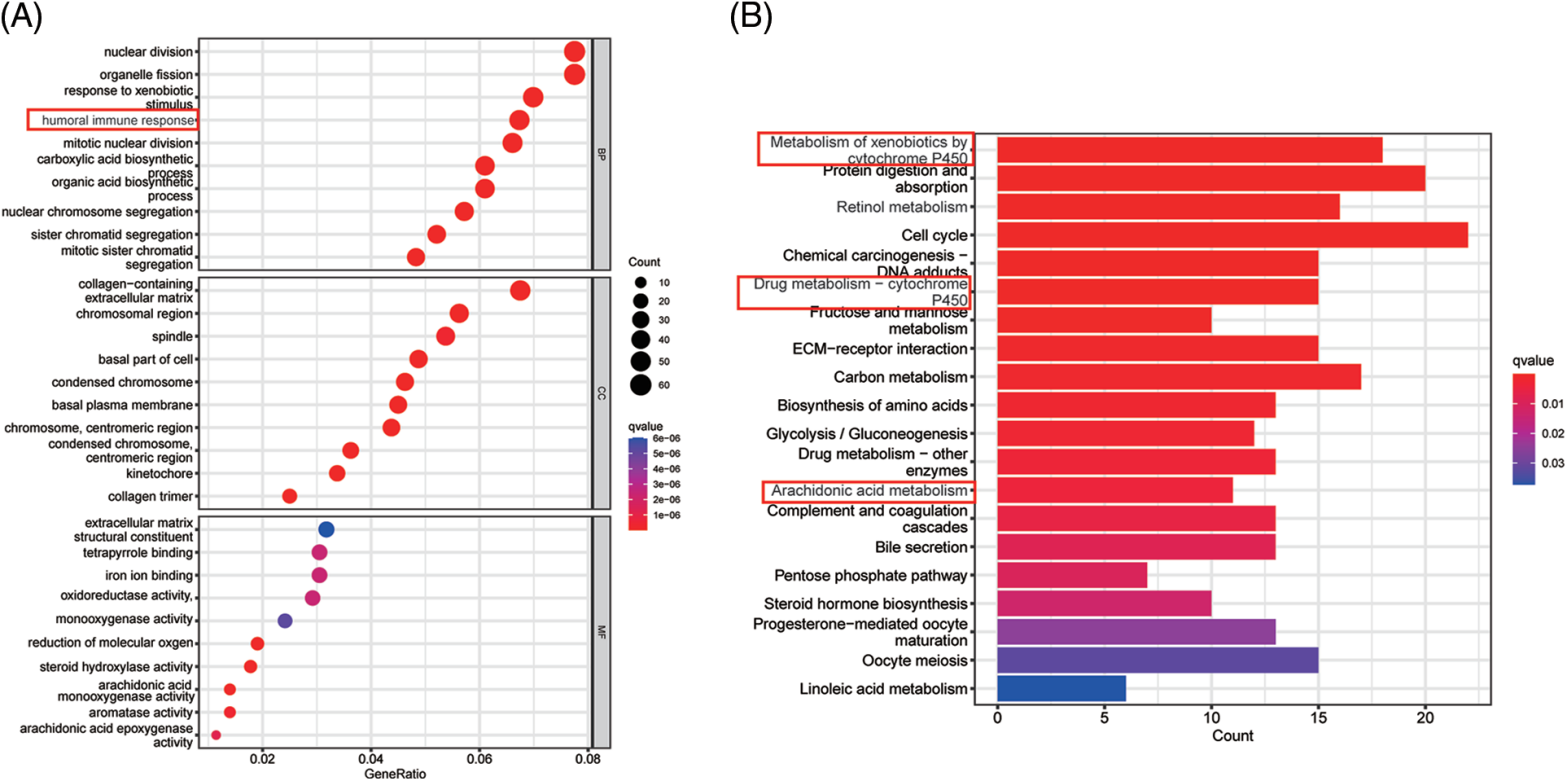
(A) GO analysis of ICGC dataset (B) KEGG analysis of ICGC dataset.

The GO is designed to find out in which GO terms differentially expressed genes are mainly enriched, molecular to biological processes, divided into three categories: molecular function (MF), cellular component (CC), biological process (BP). Many GO terms are associated with the immune system, immunoglobulin-mediated immune responses and complement activationd and other immune-related classical pathways and upregulated for biological functions. We thus further explored the impact of tumor immune cell composition and distribution on the immune status of HCC tumors (Fig. 4A). In the TCGA cohort, three types of immunocytes, including activated NK cells and M0 macrophage cells, the abundance of immune cell infiltration was significantly higher in the low PANscore group (Fig. 4B). Likewise, the expression of macrophage M0 cells was significantly higher in the low PANscore group in the ICGC cohort (Suppl. Fig. 2A). Furthermore, the KEGG pathway analysis of DEGs highlighted several metabolic pathways related to nutrition, cell cycle pathways, and cancer-related pathways (Fig. 4C). GO, and KEGG results were all validated in the ICGC cohort (Suppl. Figs. 2B and 2C). Because PANoptosis is associated with many metabolic pathways, the relationship between the eight PANRGs constructing the PANscore and 336 metabolism-related genes was analyzed (Fig. 4D). Pearson correlation analysis showed that most metabolism-related genes were associated with PANRGs, including positive and negative correlations (|r| ≥ 0.2, *p* < 0.05).

### Identification and validation of hub genes

**Figure 5:**
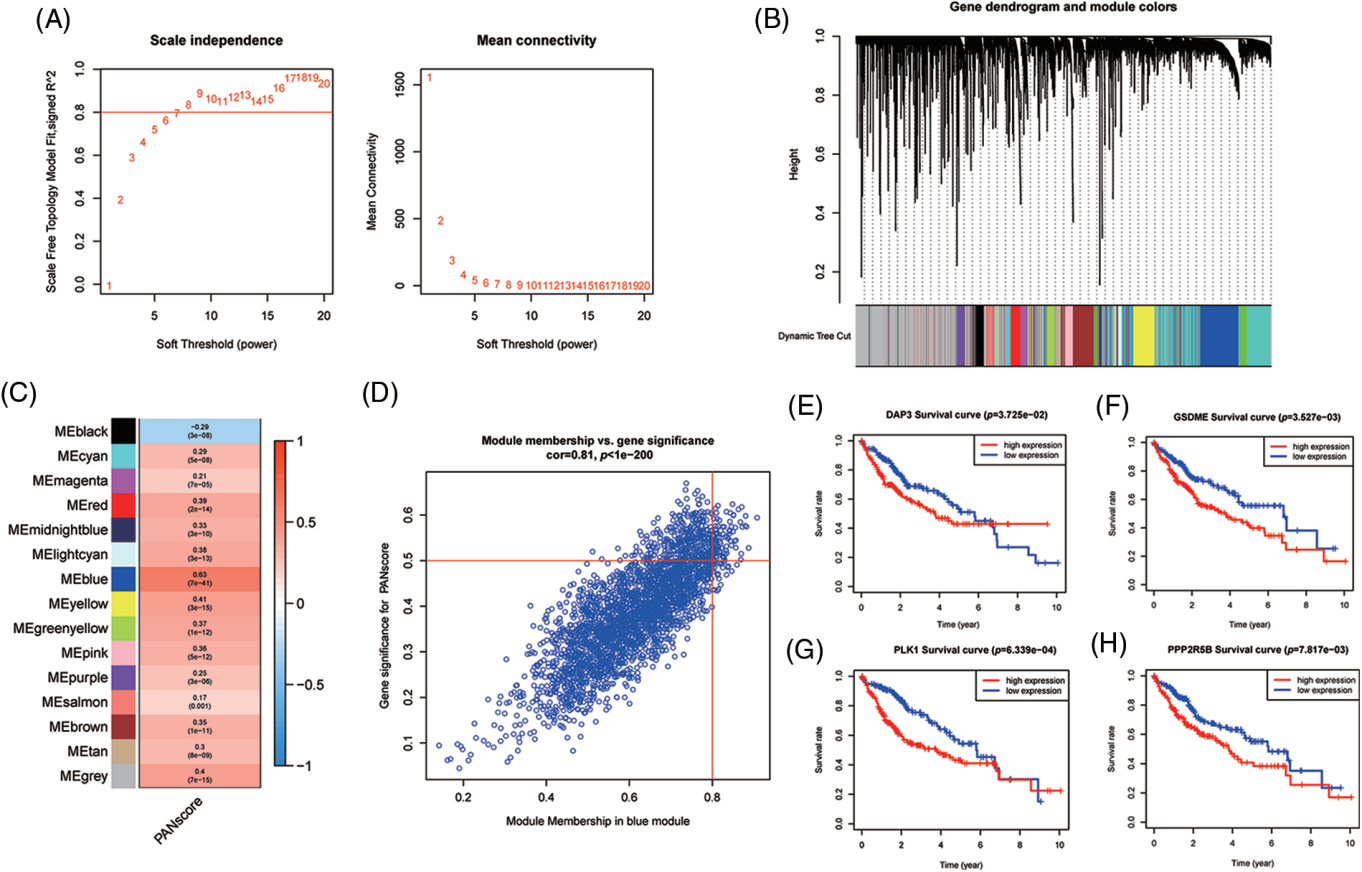
The WGCNA screening for PANoptosis-related hub genes. (A) Choose a suitable power index and construct a scale-free co-expression network. (B) The branches of the tree diagram correspond to the different gene modules. (C) The correlation coefficients and corresponding *p*-values of each gene module with PANscore are in the boxes. (D) Scatterplot of correlation between the blue module and PANoptosis gene module. (E–H) Kaplan-Meier curves for 4 hub genes.

To further analyze the hub genes in the PANoptosis model, the WGCNA method was chosen to construct key modules based on TCGA-seq data. We selected the optimal power value of 8 (Fig. 5A) to construct a scale-free coexpression network. A hierarchical clustering analysis of all samples was performed, and the results are shown in Fig. 5B. The correlation between gene modules and PANscores was calculated, and 15 gene modules associated with PANscore were identified. The blue module with the strongest positive correlation with the PANscore (coefficient = 0.63, *p* < 0.001) was selected (Figs. 5C and 5D). In the blue module, 8 PANRGs were overlapped. Finally, GSDME, PLK1, DAP3, and PPP2R5B were identified as the key PANRGs. Subsequently, Survival analysis based on RNA expression showed that DAP3, GSDME, PLK1, and PPP2R5B expression at the protein level and RNA level was associated with poor prognosis (both *p* < 0.05) (Figs. 5E–5H).

### Association between the key genes and immune infiltration

**Figure 6:**
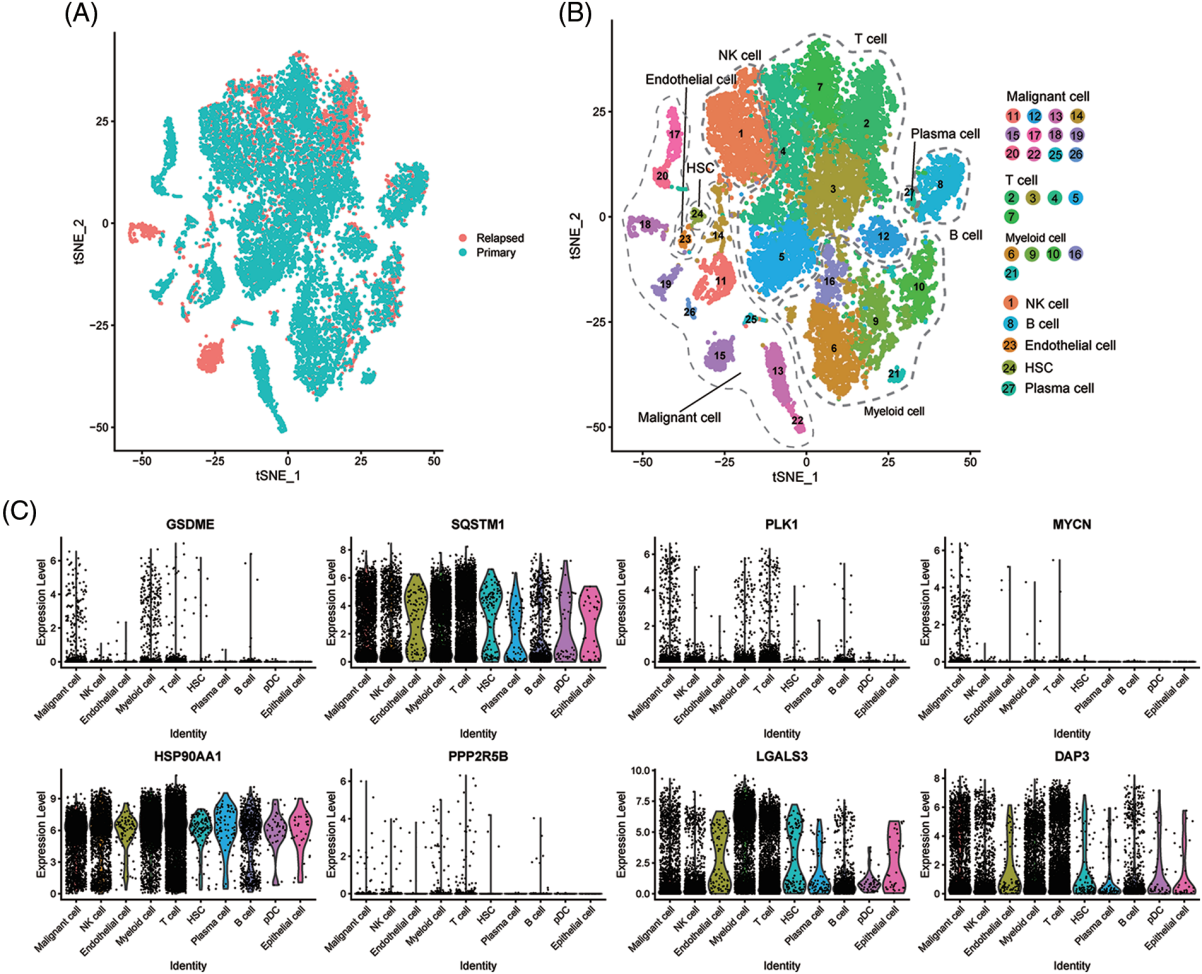
Single-cell analysis of eight PANRGs in hepatocellular carcinoma. (A) Sample source of cells. (B) t-distribution random neighbor embedding (t-SNE) plot showing annotation and color coding of HCC cell types. (C) Expression levels of 8 PANRGs in single cells.

To evaluate the expression levels of the eight genes constructed on the PANscore at the single-cell level, scRNA-Seq data from CNSA-CNP0000650 were obtained. A total of 16,498 single cells from primary- and early relapse-seq samples were assigned to single cells based on surface markers of cell types. Single cells overview of primary and early relapsed HCC samples are shown in Fig. 6A. The t-SNE method was used for the manual annotation of clusters, and we finally annotated 27 clusters corresponding to a total of 8 cell types (Fig. 6B) including malignant cells, T cells, myeloid cells, NK cells, B cells, endothelial cells, HSCs, and plasma cells. Multiple clusters can be annotated for the same cell type. Ultimately, we explored the expression of the 8 PANRGs in cell clusters, as shown in Fig. 6C. Notably, they were significantly higher in T cells, bone marrow cells, and malignant cells.

### Development and validation of the prognosis-related nomogram for HCC

**Figure 7:**
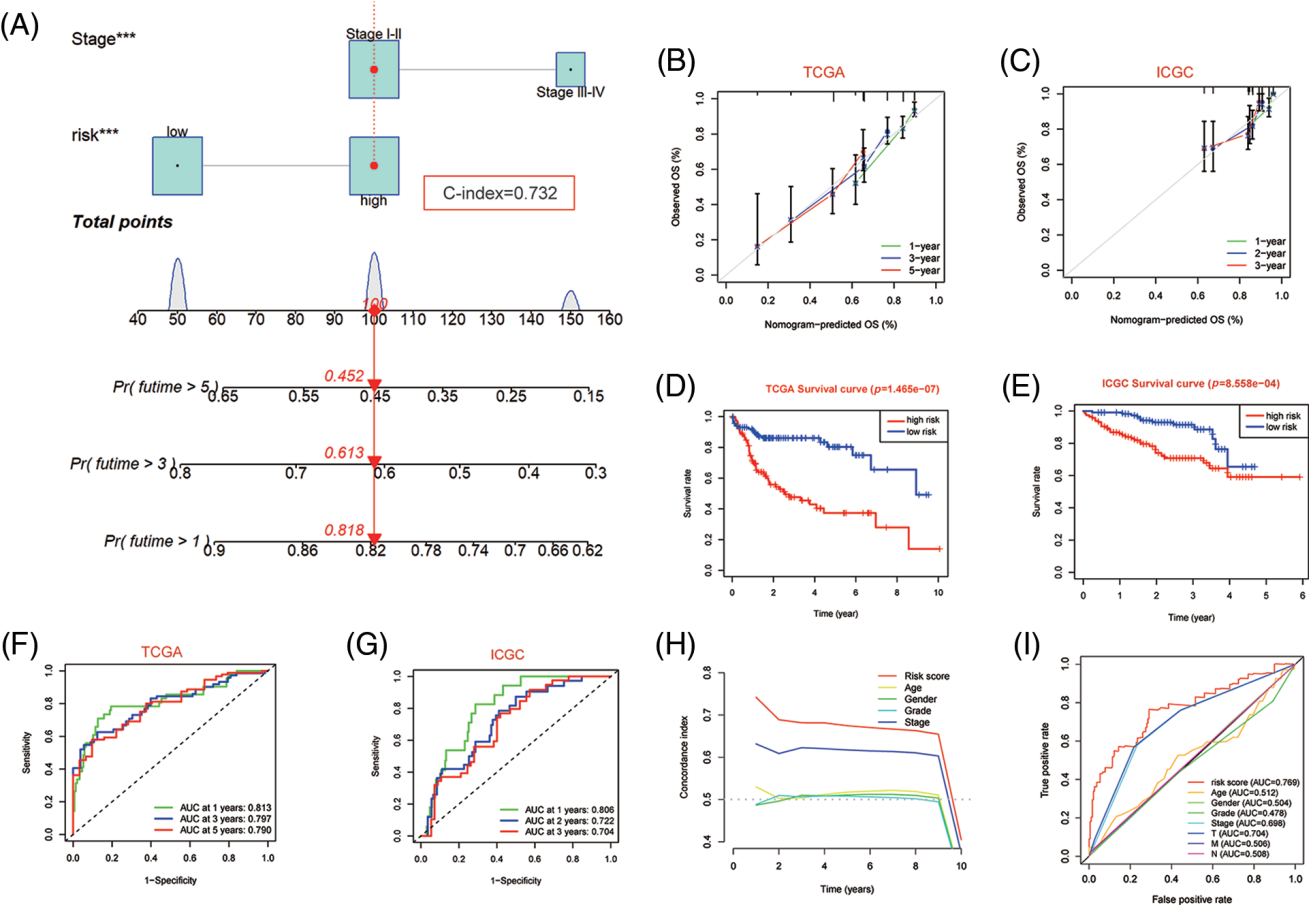
Nomogram predicting overall survival in HCC patients. (A) Nomogram constructed using clinical characteristics and PANscores from the TCGA dataset. (B) Calibration curves of Nomogram in the TCGA cohort. (D) K-M survival curves of the nomogram. (F) AUC of ROC curves showing the predictive efficiency of nomogram. (C, E, G) Validation of the predictive power of nomogram in ICGC dataset, including calibration curves, K-M analysis and ROC curves. (H) C-INDEX of different clinical indicators in TCGA datasets. (I) Multi-indicator ROC Curve in TCGA Database.

Since independent prognostic analysis demonstrated that the PANscore was an independent risk factor for HCC, we developed a novel prognostic nomogram based on the PANscore and TNM stages (Fig. 7A). The calibration plots revealed that the nomograms provided a reliable quantitative method for predicting OS in HCC patients (Figs. 7B and 7C). The K-M curves indicated that the prognosis of patients in the high-risk group was worse compared to the low-risk group (Figs. 7D and 7E). Furthermore, the probability of OS predicted by the nomogram was closely matched to the actual OS probability. In particular, the 1-year AUC value up to 0.813 and the 3-year and 5-year AUC values of 0.797 and 0.790, respectively in the TCGA cohort, and validated in the ICGC cohort (Figs. 7F and 7G).

### Validation of the occurrence of PANoptosis in HCC cells and patients and the expression of key genesThe relationship between expression of key genes and OS in HCC patients

**Figure 8:**
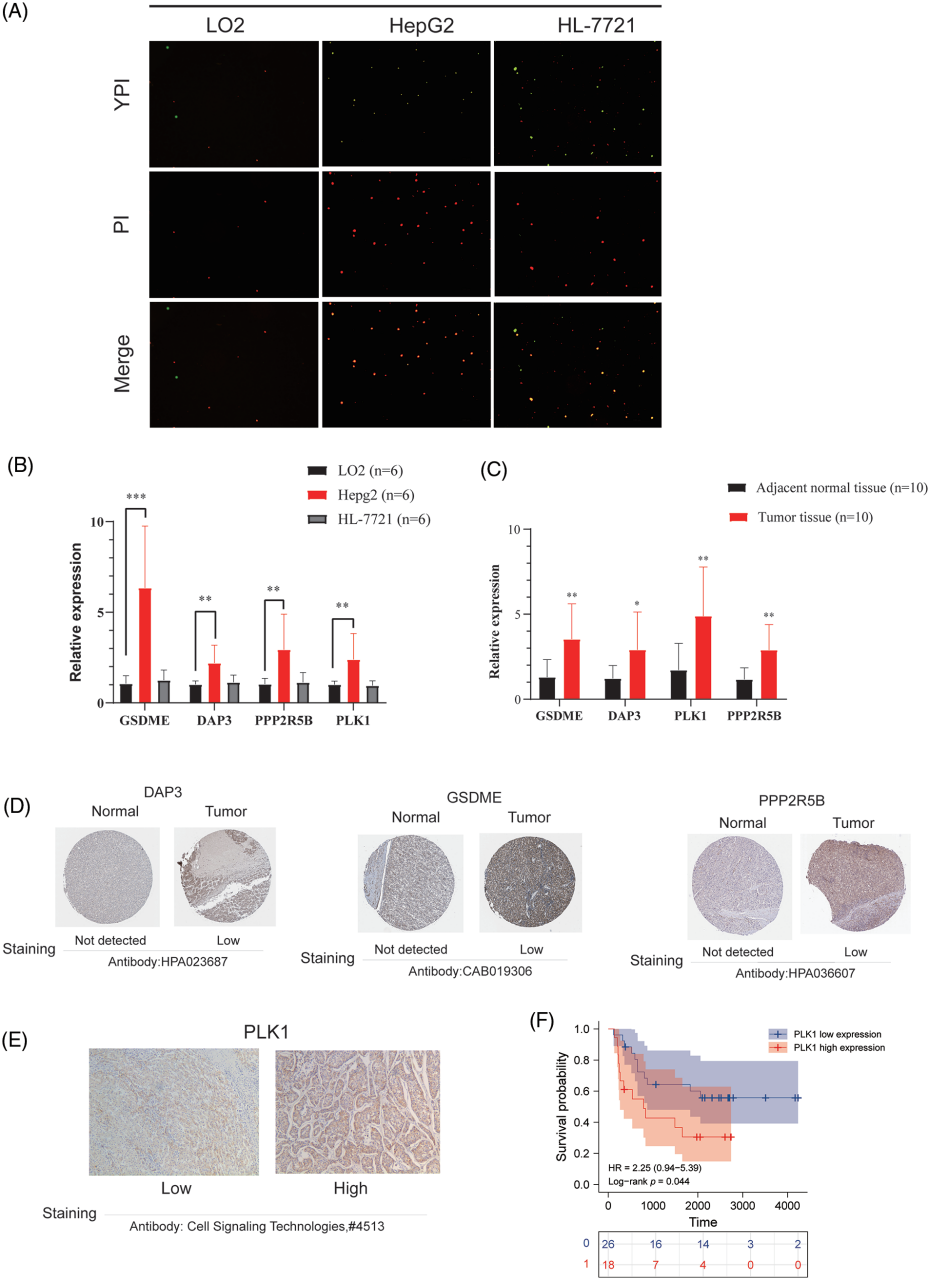
Validation of differential expression of hub genes at the cellular and protein levels. (A) Representative images showing the occurrence of PANoptosis in normal hepatocyte lines and HCC cell lines. YP1+ cells (green) may undergo apoptosis or necroptosis, PI+ cells (red) may undergo apoptosis, necroptosis, pyroptosis, or ferroptosis. (B) mRNA expression of GSDME, DAP3, PPP2R5B, and PLK1 in normal hepatocytes and HCC cell lines. (C) mRNA expression of GSDME, DAP3, PPP2R5B, and PLK1 in normal and HCC tissues. (D) Immunohistochemistry of DAP3, GSDME, and PPP2R5B in normal and tumor groups in the HPA database. (E) Immuno-histochemical images of PLK1 protein expression in hepatocellular carcinoma tumor tissues, 100×. (F) Prognostic significance of PLK1 expression in HCC patients.

To further confirm the occurrence of PANoptosis, we confirmed in normal hepatocytes (L-O2) and human hepatocellular carcinoma cells (HepG2 and HL-7721), including YP1-positive cells indicative of apoptosis or necroptosis and PI-positive cells indicative of necroptosis, pyroptosis or ferroptosis (Fig. 8A).

To evaluate the expression of four PANoptosis-related hub genes (GSDME, PLK1, DAP3, and PPP2R5B), qRT-PCR was used to quantify mRNA expression levels. The primer sequence information used for this study is shown in Table 2 Results indicated that the four PANoptosis-related hub genes in HCC tissues were upregulated. Besides, we confirmed the mRNA levels of these hub genes in human hepatocytes. As shown, the mRNA levels of GSDME, PLK1 and PPP2R5B were significantly higher in HepG2 cells than in the normal hepatocyte line L-O2, the mRNA level of DAP3 was higher in the HL-7721 cell line than in L-O2 (Fig. 8B). We also confirm the expected expression pattern of the four hub genes in human liver cancer tissues, the results showed that GSDME, DAP3, PPP2R5B, and PLK1 were overexpressed in human HCC tissues compared to paired adjacent non-tumor tissues, indicating poor prognosis (Fig. 8C).

**Table 2:** Primers used in this study

Variable	Gene name	Primer	5′-3′ Sequence	size
qPCR	GAPDH	Forward	GGAAGCTTGTCATCAATGGAAATC	168 bp
		Reverse	TGATGACCCTTTTGGCTCCC	
	PLK1	Forward	CGACTTCGTGTTCGTGGTGT	155 bp
		Reverse	GATGAATAACTCGGTTTCGGTG	
	DAP3	Forward	CCGCACCAATGAGAATGACC	138 bp
		Reverse	CTGAATGTCTTCACCTGCATCAC	
	GSDME	Forward	TACTTCTTGGTCAGTGCCCTCG	174 bp
		Reverse	CCTTTCTGTATCTTTCAGGGGAGT	
	PPP2R5B	Forward	GGACCCTCTACCAAGTCTCCAAG	187 bp
		Reverse	TCCAGACCTTGCCATAACTCCT	

To further verify the differences in protein expression of key genes, we obtained representative immunohistochemical images of GSDME, DAP3, and PPP2R5B from the HPA database (Fig. 8D). The expression of PLK1 in 46 HCC tissues was then examined by immunohistochemistry. PLK1 protein expression levels were significantly increased in tumor tissues (Fig. 8E). Then, the prognostic impact of PLK1 on the OS of HCC patients was evaluated by K-M. As shown in Fig. 8F, PLK1 resulted in a significant survival difference in the 46 HCC samples (*p* = 0.044). The baseline information for these patients is summarized in Table 3.

**Table 3:** Clinical information of the study patients

Characteristics	Number (N = 46)
Age (years)	57.19 ± 8.778
Sex	
Male	33 (76.7%)
Female	11 (23.3%)
Smoking	
Yes	23 (53.5%)
No	20 (46.5%)
Metastasis	
Yes	5 (11.6%)
No	38 (88.4%)
Family history	
Yes	4 (9.3%)
No	39 (90.7%)

### Effects of DAP3, GSDME, PLK1, and PPP2R5B knockdown on the growth of pan-cancer cell lines and HCC cells

**Figure 9:**
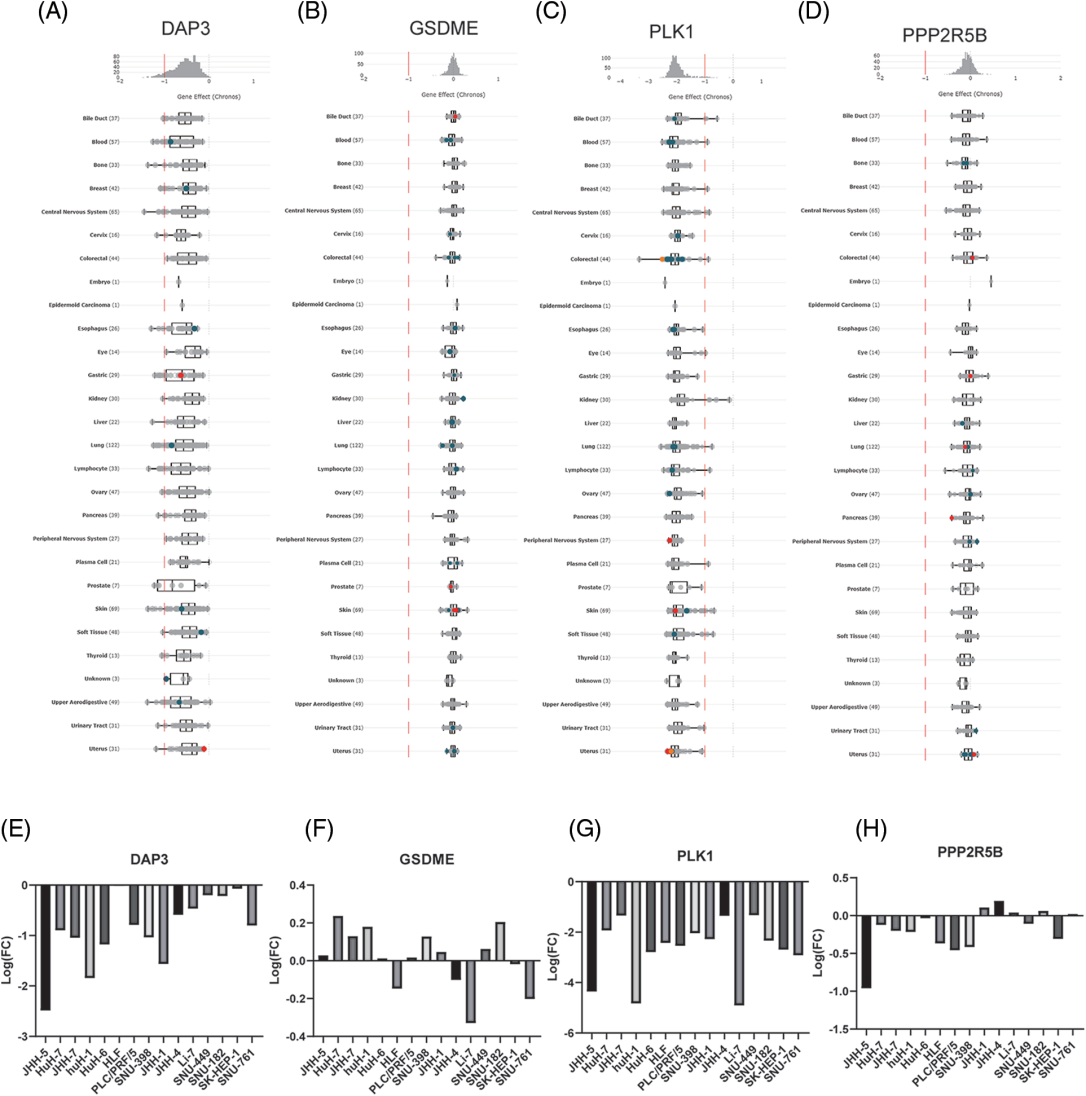
The role of gene knockout on cell growth. (A) Pan-cancer analysis of the effect of knockdown of four hub genes on cell lines growth. (B) Effect of GSDME, DAP3, PPP2R5B, and PLK1 knockdown on the proliferation of HCC cells.

In this study, the effects of DAP3, GSDME, PLK1, and PPP2R5B knockdown on cancer cell growth were analyzed by DepMap, a cancer survival-dependent gene database. log2(FC) < −1 indicates that knockdown inhibits the growth of cancer cell lines. The results indicated that DAP3 and PLK1 knockdown could affect the growth of a variety of tumor cells, including liver cancer, colorectal cancer, and gastric cancer as shown in Figs. 9A–9D. Data from screening cancer growth-related genes by mining the genome-wide CRISPR-Cas9 knockdown database showed that DAP3 gene knockdown could inhibit the growth of JHH-5, HuH-1, SNU398, and other liver cancer cell lines. Meanwhile, PLK1 knockdown inhibited the growth of JHH-5, HuH-1, SK-HEP-1, and other HCC cell lines. The impact of GSDME and PPP2R5B knockdown on HCC cells was shown in Figs. 9E–9H.

## Discussion

PANoptosis kills cancer cells and inhibits tumor growth by inducing cell death through TNF-α and IFN-γ [12], thus contributing to the treatment and intervention of cancer [25,26]. PANoptosis was regulated by a cytoplasmic polyprotein complex called PANoptosome, which can simultaneously involve three programmed cell death patterns, including pyroptosis, apoptosis, and necroptosis. Meanwhile, the PANoptosome was made up of the key regulators of apoptosis caspase-8, the pyroptosis components caspase-1 and caspase-11, and the necroptosis components RIPK1 and RIPK3, which integrate the three programmed cell death mechanisms into a unified mechanism. Previous studies showed that most cancer cells can resist cell death and maintain cell growth [27], this makes it possible for PANoptosis to target cancer prevention and treatment [28]. The exploration of tumor and PCD mechanisms is currently the subject of intense research [29–31], and the potential association between HCC and PANoptosis remains elusive.

In this study, we used a gene list including 274 PANRGs, and LASSO-Cox regression analysis was used to screen a risk prognostic model PANscore consisting of 8 PANoptosis-related genes (including DAP3, LGALS3, PPP2R5B, HSP90AA1, MYCN, PLK1, SQSTM1, and GSDME) to support the prediction of HCC prognosis. Notably, eight genes associated with three PCDs were involved in the process of PANscore, which further confirmed the complex crosstalk between PCD pathways when PANoptosis occurs. In addition, correlation analysis between PANRGs showed a significant correlation between the 3 PCDs. Subsequently, we developed a nomogram [32] using a combination of independent prognostic indicators PANscore and TMN, which performed better than the earlier single indicators and was well calibrated with good agreement between external validation and the training set.

The eight PANRGs that construct the PANscore can be roughly classified into three types, apoptosis (DAP3, LGALS3, and PPP2R5B), Necroptosis (HSP90AA1, MYCN, SQSTM1, and PLK1), and pyroptosis (GSDME). Among them, death-associated protein 3 (DAP3) is a molecule that plays an important role in the control of apoptosis [19]. as a subunit of mitochondria, DAP3 is involved in the process of detachment from extracellular matrix-induced anoikis-induced apoptosis [33] and mediates IFN-induced apoptosis (interferon-γ), tumor necrosis factor receptor (TNF-α), Fas ligand (FasL) and TNF-related apoptosis-inducing ligand (TRAIL) to induce apoptosis [24]. Meanwhile, DAP3 is at the location of several important cellular pathways in tumorigenesis and has been shown to be expressed at elevated levels in aggressive glioblastoma cells and glioma cell lines with an induced migration phenotype [34]; survival analysis by Gera et al. [35] in a clinical study of a cohort of 127 breast cancer patients showed higher survival rates in the highly transcribed group. Galactose lectin-3 (LGALS3, also known as galectin-3), a member of the galectin protein family, is one of the most widely studied galactose lectins in cancer and is involved in a variety of cellular activities including apoptosis, cell migration, proliferation, and angiogenesis [36–38]. It is able to participate in apoptosis through a conserved carbohydrate recognition domain (CRD) that allows the binding of β-galactose-containing glycocalyxes [29]. Pratima et al. elaborated the regulatory role of LGALS3 in tumor stem cells (CSCs)-related phenotypes and signaling pathways [39], and it has also been shown that galactose lectin-3 is an important regulator of lung adenocarcinoma progression [40]. PPP2R5B is a subunit encoding protein phosphatase 2A (PP2A), which is associated with insulin resistance [41].PP2A is widely recognized as a tumor suppressor, studies have shown that low expression of PPP2R5B improves survival [42]. The mechanism of action of PPP2R5B in HCC is still unclear.HSP90AA1 belongs to the heat shock protein (HSP90) family, and many of its regulated client proteins are proto-oncogene products or important signal transduction factors in tumorigenesis and are closely related to tumor development [43,44]. It inhibits cancer cell proliferation and increases necrosis [45,46].MYCN proto-oncogenes regulate cell growth, apoptosis and differentiation and are associated with tumor aggressiveness and chemoresistance [47]. Specifically, MYCN overexpression promotes cell cycle progression, inhibits cytotoxic T cell infiltration, and accelerates lung cancer progression [48]. And one study found that MYCN is overexpressed in HCC cell lines and clinical samples from HCC patients, and also inhibits HCC cell growth and invasion by knocking down the gene [49]. p62/SQSTM1 is the protein responsible for mitosis, and the mitotic signaling pathway appears to be impaired in cancer [50,51]. Mitosis acts as a switch that regulates metabolism and can promote cancer cell survival [52].

Polo-like kinase 1 (PLK1) is an important regulator of the cell cycle [53], and PLK1 expression is significantly higher in HCC tissues than in normal liver tissues [54]. Inhibition of PLK1 leads to cellular G2/M blockade, inhibition of proliferation, and downregulation of Survivin expression to promote apoptosis, making it possible to use PLK1 inhibitors as a potential therapeutic tool to treat cancer [55]. Lin et al. showed that PLK1 inhibition significantly prevented liver tumorigenesis in transgenic mice, primary cells, and HCC [56], proposing a new strategy for ubiquitinylase-PLK1-targeted treatment of hepatocellular carcinoma. Gasdermin E (GSDME) is the central molecule regulating the process of scorching and necrosis. Its mechanism of action is the specific cleavage by caspase-3 at its junction, which generates a GSDME-n fragment that penetrates the cell membrane and thus induces scorch death [57]. In different cancers, GSDME has different expression levels and functions, and even the subcellular localization of GSDMD affects cancer progression and immune response. In cancer cells with low GSDME expression, Caspase3 tends to induce apoptosis. By staining hepatocellular carcinoma cells and normal hepatocytes with YO-PRO-1/PI fluorescence, we found that apoptosis and necrosis were rarely observed in normal hepatocytes, whereas extensive apoptosis, scorching and necrosis occurred simultaneously in hepatocellular carcinoma cells. Therefore, it can be assumed that pan-apoptosis occurred in HCC. Growing evidence suggests that tumor-infiltrating leukocytes (TILs), including B cells, effector T cells, and memory T cells, play a critical role in the clinical management of HCC patients [58]. In previous studies, macrophages, neutrophils, and Treg cells were shown to promote tumor progression [59–61]. The poor clinical prognosis of HCC patients is associated with NK-cell dysfunction [62]. GO and KEGG analysis based on pathways reveal immune cell infiltration and metabolism-related pathways that partly play a role in PANoptosis-related processes. Unexpectedly, DEGs are enriched in many biological processes and pathways related to immunity. Thus, it is reasonable to assume that PANoptosis is closely related to tumor immunity. We then used the CIBERSORT algorithm to quantify tumor-infiltrating immune cells and found that M0 macrophage cells and NK cells were significantly higher in the high PANscore group. Subsequently, to further analyze the PANoptosis pattern of HCC at the single-cell level, we found that the expression of prognostic PANRGs was significantly higher in T cells. HSP90AA1, SQSTM1, LGALS3, and DAP3 were significantly expressed in T cells, HSCs, myeloid cells, and other cells. GSDME and PLK1 showed distinct expression in T cells and myeloid cells.

CIRBERSORT is a method to characterize the cellular composition of complex tissues through gene expression profiles, enabling large-scale analysis of RNA mixtures, deconvolution of expression matrices of human immune cell subtypes based on the principle of linear support vector regression (linear support vector regression), by estimating relative subpopulations of RNA transcripts to accurately identify cell types of GEPs from complex tissues to find biomarkers and therapeutic targets [20]. Subsequently, we quantified tumor-infiltrating immune cells using the CIBERSORT algorithm and found a higher abundance of M0 macrophages and NK cells in the high PANscore group. Liver macrophages play a central role in the pathogenesis of liver disease (hepatic macrophages play a central role in the pathogenesis of liver disease), while NK cells are a unique cytotoxic lymphocyte that plays a critical role in fighting tumors and infections as an important component of the anti-tumor natural immune system, and the phenotype and function of NK cells have been demonstrated in patients with liver cancer, where tumor infiltration and circulating NK cell recurrence rate is positively correlated with survival benefit in HCC and has prognostic significance, and NK cell dysfunction is closely associated with HCC progression [63]. Single-cell RNA sequencing (scRNA-seq) technology allows the resolution of gene expression at single-cell resolution, dramatically revolutionizing transcriptomic studies, and its application in profiling the tumor microenvironment has brought important insights into the biology of tumor-infiltrating immune cells, including their heterogeneity, dynamics, and potential role in disease progression and response to immune checkpoint inhibitors and other immunotherapies (PMID: 28787399). Subsequent to further analysis of HCC explored at the single cell level, we found that HSP90AA1, SQSTM1, LGALS3 and DAP3 were significantly expressed in T cells, hematopoietic stem cells, bone marrow cells and other cells. gSDME and PLK1 showed differential expression in T cells and bone marrow cells.

Nomogram is a method that can predict the risk or prognosis of each patient’s disease by combining multiple clinical indicators and plotting different indicators into different lines [64]. In one study, the predictive nomogram technique based on radiomic and clinical indicators could better predict TACE response in intermediate to advanced HCC and could be further used as an aid to clinical prognosis [65]. A study showed that nomograms combining clinical radiological risk factors and radiological features of hepatobiliary phase images could better predict HCC patients [66] The main advantage of nomograms is that they allow individualized risk assessment based on patient or disease characteristics. Many nomograms have been used for individualized prognostic prediction of different cancers [66–68]. In our study, we constructed a prognostic column line graph combining clinical features and pan-apoptotic features. More importantly, our nomogram predicted prognosis with higher accuracy and clinical value than the conventional TNM staging system.

Furthermore, four key genes (DAP3, GSDME, PPP2R5B, and PLK1) were screened from the PANscore model gene members, some of them have been reported to be associated with poor prognosis in HCC. Previous studies have suggested that GSDME is overexpressed in HCC [13] and high expression of PPP2R5B was significantly associated with poor OS [69]. However, since the relationship between DAP3, GSDME, PPP2R5B, and PLK1 expression and HCC has not been previously reported, we performed the qRT-PCR analysis in LO2, HepG2, and HL-7721 cells and showed that GSDME, PPP2R5B, and PLK1 expression was upregulated in HCC cell lines. Similarly, the expression of DAP3, GSDME, PPP2R5B, and PLK1 was significantly higher in 15 HCC tissues. IHC showed that high expression of GSDME, PPP2R5B, and PLK1 was associated with poor prognosis in HCC patients.

Depmap database was used to explore genome-wide knockout library screening data, and pan-cancer analysis of DAP3, PPP2R5B, PLK1, and GSDME genes on cancer cell growth. The results showed that the knockdown or silencing of DAP3 and PLK1 genes could inhibit the growth of most HCC cells and many other cancer cells.

To our knowledge, this is the first study to report the molecular, and immune characteristics associated with PANoptosis in HCC. Our study provides new insights into PANoptosis in HCC, and subsequent studies are expected to illuminate more potential molecular mechanisms of PANoptosis, thereby providing more evidence for cancer prevention and treatment.

Inevitably, this study still has some shortcomings, such as the sample size included is relatively small, as well as the adoption of retrospective research methods, which cannot predict cancer occurrence and early diagnosis. Later, we will increase the model credibility by continuing to collect HCC samples and relevant prognostic information and establishing cohorts for prospective s follow-up studies.

## Conclusions

Here, we obtained four key genes and an independent validated PANscore by comprehensive analysis. The PANscore contributes to OS prediction in HCC patients. Our PANscore is expected to be a clinically useful tool for individualized treatment and a specific indicator to assess prognosis in HCC.

## Supplementary Materials

Supplementary Table 1 Summary table of PANnoptosis genes

## Data Availability

Data available on request from the authors.

## Abbreviations

PCD: Programmed cell death
HCC: Hepatocellular carcinoma
PANRGs: PANoptosis-related genes
LASSO: Least absolute shrinkage and selection operator
WGCNA: Weighted gene coexpression network analysis
IHC: Immunohistochemistry
OS: Overall survival
K-M: Kaplan-Meier
qRT-PCR: quantitative real-time PCR
TCGA: The Cancer Genome Atlas
ICGC: The International Cancer Genome Consortium
CNGBdb: The China National GeneBank DataBase
FPKM: Fragments per kilobase million
RNA-seq: RNA-sequencing
GO: Gene Ontology
KEGG: Kyoto Encyclopedia of Genes and Genomes
ssGSEA: Single-sample GSEA
scRNA: Single-cell RNA
YP1: YO-PRO-1
PI: Propidium iodide
AUC: Area under the curve
GSEA: Gene set enrichment analysis
DAP3: Death-associated protein 3
TNF-α: Tumor necrosis factor receptor
FasL: Fas ligand
TRAIL: TNF-related apoptosis-inducing ligand
CRD: Conserved carbohydrate recognition domain
PP2A: Phosphatase 2A
HSP90: Heat shock protein
PLK1: Polo-like kinase 1
GSDME: Gasdermin E
TILs: Tumor-infiltrating leukocytes
