# Supplementary material for: Construction of the panoptosis-related gene model and characterization of tumor microenvironment infiltration in hepatocellular carcinoma
Source: Oncol Res. 2023 Jun 27;31(4):569–90. doi: 10.32604/or.2023.028964 (PMC10319585; doi:10.32604/or.2023.028964)
Supplement: Supplementary Table 1 [file OncolRes-31-28964-s001.pdf]

Supplementary Table 1 Summary table of PANnoptosis genes.

Gene	Type
ADD1	apoptosis
AIFM1	apoptosis
AIFM3	apoptosis
AKT3	apoptosis
ANKH	apoptosis
ANXA1	apoptosis
APAF1	apoptosis
APPL1	apoptosis
ATF3	apoptosis
AVPR1A	apoptosis
BCAP31	apoptosis
BCL10	apoptosis
BCL2L1	apoptosis
BCL2L10	apoptosis
BCL2L2	apoptosis
BGN	apoptosis
BID	apoptosis
BIK	apoptosis
BIRC3	apoptosis
BMF	apoptosis
BMP2	apoptosis
BNIP3L	apoptosis
BOK	apoptosis
BRCA1	apoptosis
BTG2	apoptosis
BTG3	apoptosis
CASP10	apoptosis
CASP2	apoptosis
CASP7	apoptosis
CAV1	apoptosis
CCNA1	apoptosis
CCND1	apoptosis
CCND2	apoptosis
CD14	apoptosis
CD2	apoptosis
CD38	apoptosis
CD44	apoptosis
CD69	apoptosis
CDC25B	apoptosis
CDK2	apoptosis
CDKN1A	apoptosis
CDKN1B	apoptosis
CHUK	apoptosis
CLU	apoptosis
CRADD	apoptosis
CREBBP	apoptosis
CTH	apoptosis
CTNNB1	apoptosis
DAP	apoptosis

DAP3	apoptosis
DCN	apoptosis
DDIT3	apoptosis
DFFA	apoptosis
DFFB	apoptosis
DNAJA1	apoptosis
DNAJC3	apoptosis
DNM1L	apoptosis
DPYD	apoptosis
E2F1	apoptosis
EBP	apoptosis
EGR3	apoptosis
EMP1	apoptosis
ENO2	apoptosis
ERBB2	apoptosis
ERBB3	apoptosis
EREG	apoptosis
ETF1	apoptosis
F2	apoptosis
F2R	apoptosis
FDXR	apoptosis
FEZ1	apoptosis
GADD45A	apoptosis
GADD45B	apoptosis
GCH1	apoptosis
GNA15	apoptosis
GPX1	apoptosis
GPX3	apoptosis
GSN	apoptosis
GSR	apoptosis
GSTM1	apoptosis
GUCY2D	apoptosis
H1F0	apoptosis
HGF	apoptosis
HMGB2	apoptosis
HMOX1	apoptosis
HSPB1	apoptosis
IER3	apoptosis
IFITM3	apoptosis
IFNB1	apoptosis
IFNGR1	apoptosis
IGF1	apoptosis
IGF2R	apoptosis
IGFBP6	apoptosis
ISG20	apoptosis
JUN	apoptosis
KRT18	apoptosis
LGALS3	apoptosis
LMNA	apoptosis
LUM	apoptosis
LY96	apoptosis
MADD	apoptosis
MCL1	apoptosis

MGMT	apoptosis
MMP2	apoptosis
NEDD9	apoptosis
NEFH	apoptosis
PAK1	apoptosis
PDCD4	apoptosis
PDGFRB	apoptosis
PEA15	apoptosis
PLAT	apoptosis
PLCB2	apoptosis
PLPPR4	apoptosis
PMAIP1	apoptosis
PPP2R5B	apoptosis
PPP3R1	apoptosis
PPT1	apoptosis
PRF1	apoptosis
PSEN1	apoptosis
PSEN2	apoptosis
PTK2	apoptosis
RARA	apoptosis
RELA	apoptosis
RETSAT	apoptosis
RHOB	apoptosis
RHOT2	apoptosis
RNASEL	apoptosis
ROCK1	apoptosis
SAT1	apoptosis
SATB1	apoptosis
SC5D	apoptosis
SLC20A1	apoptosis
SMAD7	apoptosis
SOD1	apoptosis
SOD2	apoptosis
SPTAN1	apoptosis
TAP1	apoptosis
TFDP1	apoptosis
TGFB2	apoptosis
TGFBR3	apoptosis
TICAM1	apoptosis
TIMP1	apoptosis
TIMP2	apoptosis
TIMP3	apoptosis
TNFRSF12A	apoptosis
TOP2A	apoptosis
TP73	apoptosis
TSPO	apoptosis
TXNIP	apoptosis
UACA	apoptosis
UNC5B	apoptosis
VDAC2	apoptosis
WEE1	apoptosis
XIAP	apoptosis
YWHAE	apoptosis

YWHAG	apoptosis
ALK	Necroptosis
APP	Necroptosis
ATRX	Necroptosis
AXL	Necroptosis
BACH2	Necroptosis
BCL2	Necroptosis
BCL2L11	Necroptosis
BNIP3	Necroptosis
BRAF	Necroptosis
CD40	Necroptosis
CDKN2A	Necroptosis
CFLAR	Necroptosis
CYLD	Necroptosis
DDX58	Necroptosis
DIABLO	Necroptosis
DNMT1	Necroptosis
EGFR	Necroptosis
FADD	Necroptosis
FAS	Necroptosis
FASLG	Necroptosis
FLT3	Necroptosis
GATA3	Necroptosis
HAT1	Necroptosis
HDAC9	Necroptosis
HSP90AA1	Necroptosis
HSPA4	Necroptosis
ID1	Necroptosis
IDH1	Necroptosis
IDH2	Necroptosis
IPMK	Necroptosis
ITPK1	Necroptosis
KLF9	Necroptosis
LEF1	Necroptosis
MAP3K7	Necroptosis
MAPK8	Necroptosis
MLKL	Necroptosis
MPG	Necroptosis
MYC	Necroptosis
MYCN	Necroptosis
OTULIN	Necroptosis
PANX1	Necroptosis
PLK1	Necroptosis
RIPK1	Necroptosis
RIPK3	Necroptosis
RNF31	Necroptosis
SIRT1	Necroptosis
SIRT2	Necroptosis
SIRT3	Necroptosis
SLC39A7	Necroptosis
SPATA2	Necroptosis
SQSTM1	Necroptosis
STAT3	Necroptosis

STUB1	Necroptosis
TARDBP	Necroptosis
TERT	Necroptosis
TLR3	Necroptosis
TNFRSF1A	Necroptosis
TNFRSF1B	Necroptosis
TNFRSF21	Necroptosis
TNFSF10	Necroptosis
TRAF2	Necroptosis
TRIM11	Necroptosis
TSC1	Necroptosis
USP22	Necroptosis
ZBP1	Necroptosis
AIM2	pyroptosis
BAK1	pyroptosis
BAX	pyroptosis
CASP1	pyroptosis
CASP3	pyroptosis
CASP4	pyroptosis
CASP5	pyroptosis
CASP6	pyroptosis
CASP8	pyroptosis
CASP9	pyroptosis
CHMP2A	pyroptosis
CHMP2B	pyroptosis
CHMP3	pyroptosis
CHMP4A	pyroptosis
CHMP4B	pyroptosis
CHMP4C	pyroptosis
CHMP6	pyroptosis
CHMP7	pyroptosis
CYCS	pyroptosis
ELANE	pyroptosis
GPX4	pyroptosis
GSDMA	pyroptosis
GSDMB	pyroptosis
GSDMC	pyroptosis
GSDMD	pyroptosis
GSDME	pyroptosis
GSMDC	pyroptosis
GZMA	pyroptosis
GZMB	pyroptosis
HMGB1	pyroptosis
IL18	pyroptosis
IL1A	pyroptosis
IL1B	pyroptosis
IL6	pyroptosis
IRF1	pyroptosis
IRF2	pyroptosis
NLRC4	pyroptosis
NLRP1	pyroptosis
NLRP2	pyroptosis
NLRP3	pyroptosis

NLRP6	pyroptosis
NLRP7	pyroptosis
NOD1	pyroptosis
NOD2	pyroptosis
PJVK	pyroptosis
PLCG1	pyroptosis
PRKACA	pyroptosis
PYCARD	pyroptosis
SCAF11	pyroptosis
TIRAP	pyroptosis
TNF	pyroptosis
TP53	pyroptosis
TP63	pyroptosis
